# Tailoring Anesthesia for Cardiac Amyloidosis: A Case Report Highlighting Perioperative Challenges

**DOI:** 10.7759/cureus.96512

**Published:** 2025-11-10

**Authors:** Ana Sofia M Fernandes, Carla Cavaleiro, André Pombo

**Affiliations:** 1 Anesthesiology, Unidade Local de Saúde de Santo António, Porto, PRT

**Keywords:** cervical spine fracture, orthopedic anesthesia, tailored management, transthyretin amyloid cardiomyopathy, wild-type transthyretin amyloidosis

## Abstract

Cardiac amyloidosis is a cause of restrictive cardiomyopathy, posing significant challenges in perioperative care. We report the case of an 85-year-old male with wild-type transthyretin amyloidosis (ATTRwt) undergoing urgent anterior cervical arthrodesis, following a traumatic cervical spine injury (C5-C6 fracture-dislocation and C2-C7 compressive hematoma). Preoperative evaluation revealed marked biventricular dysfunction (left ventricular ejection fraction 27%), atrial fibrillation, conduction abnormalities, and other comorbidities, including Parkinson’s disease and chronic kidney disease. A tailored anesthetic approach was adopted, incorporating balanced general anesthesia, advanced hemodynamic monitoring, such as cardiac output assessment, and awake fiberoptic intubation to mitigate cardiovascular and airway risks. Anticoagulation was reversed with idarucizumab, and perioperative hemodynamic instability was managed with vasopressors and dynamic fluid responsiveness assessment. This case highlights the complexity of anesthetic management in amyloidosis, particularly with cardiac involvement, which requires careful anesthetic titration to minimize vasodilation and myocardial depression and avoidance of beta-blockers and calcium channel blockers. Furthermore, the authors highlight the perioperative hematologic, metabolic, and electrolyte disorders that may arise from disease and cardiac-target therapies such as tafamidis and sodium-glucose cotransporter-2 inhibitors (SGLT2 inhibitors). We emphasize the importance of individualized planning, multidisciplinary coordination, and vigilant perioperative monitoring to optimize outcomes in this high-risk population.

## Introduction

Amyloidosis encompasses a spectrum of disorders characterized by the extracellular deposition of insoluble amyloid fibrils throughout various tissues and organs [1-2]. Cardiac amyloidosis is a rare and often disregarded cause of cardiac dysfunction. Fibrils composed of monoclonal immunoglobulin light chains (AL) or transthyretin (ATTR), either in their hereditary or acquired form, currently account for the vast majority of cardiac amyloidosis types [1].

Cardiac amyloidosis typically appears within a constellation of systemic clinical findings such as musculoskeletal, neurologic, gastrointestinal, and renal manifestations [1-3]. Wild-type transthyretin amyloidosis (ATTRwt) is commonly characterized by musculoskeletal findings, such as a history of carpal tunnel syndrome, hip or knee replacement, prior shoulder surgery (spontaneous biceps tendon rupture), or spinal stenosis. Alternatively, AL and hereditary ATTR types are associated with macroglossia, nephropathy, polyneuropathy, autonomic dysfunction, spontaneous bruising, and hepatomegaly [1,3]. Anesthesia providers must understand the multi-organ implications of amyloid infiltration to appropriately tailor anesthetic management and ensure perioperative safety. The authors describe the anesthetic approach for an anterior cervical arthrodesis in a patient with severe cardiac dysfunction due to ATTRwt.

## Case presentation

**Figure 1:**
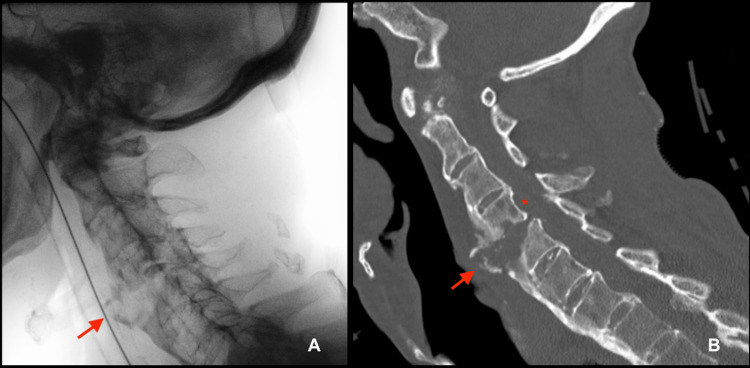
X-ray (A) and computed tomography scan (B) of the spinal fracture-dislocation Arrow: C5-C6 fracture-dislocation; Asterisk: C2-C7 compressive hematoma

An 85-year-old male with Parkinson’s disease presented to the Emergency Department following a fall, reporting bilateral motor-sensory deficits in his upper limbs. He had a history of impaired mobility and recurrent falls. An X-ray (Figure 1A) and computed tomography scan (Figure 1B) of the spine showed a C5-C6 fracture-dislocation and a C2-C7 compressive hematoma.

**Figure 2:**
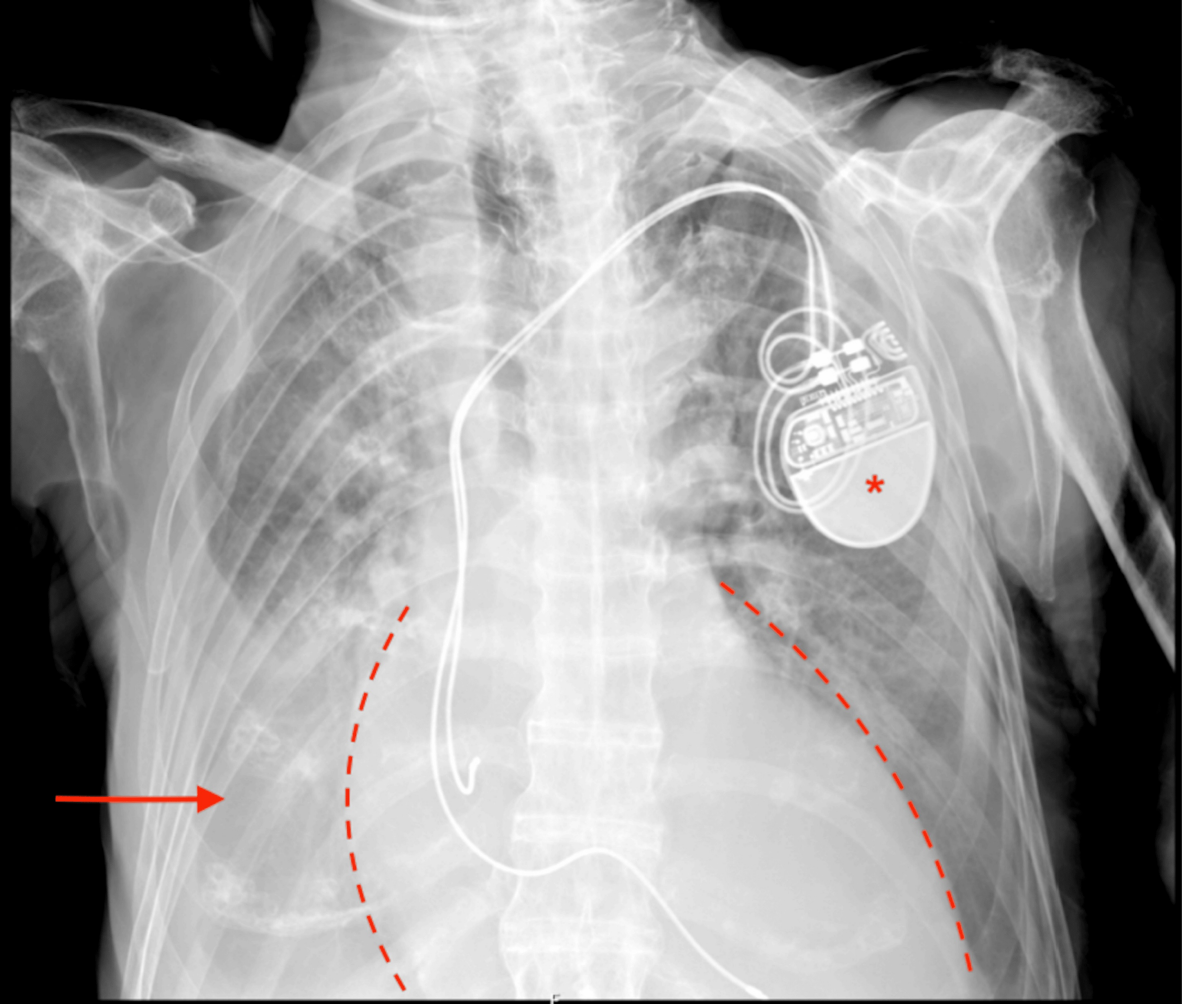
Preoperative chest X-ray in the supine position Arrow: right-sided pleural effusion; Asterisk: double-chamber DDI pacemaker; Dashed line: enlarged cardiac silhouette

Preoperative evaluation revealed echocardiographic evidence of severe left ventricular systolic and diastolic dysfunction (ejection fraction 27%) with right ventricular dysfunction, mild aortic stenosis, and moderate pulmonary hypertension, as well as atrial fibrillation and bilateral pleural effusions, in the context of ATTRwt cardiac amyloidosis. The patient’s conduction system disease, in conjunction with a hereditary SCN5A gene mutation linked to Brugada syndrome, warranted the implantation of a dual-chamber DDI pacemaker (Figure 2). According to the 2021 CKD-EPI creatinine equation [4], the patient presented stage one chronic kidney disease (eGFR 90 mL/min/1.73m^2^). Ambulatory treatment included tafamidis, dabigatran, dapagliflozin, furosemide, fluoxetine, esomeprazole, benserazide plus levodopa, and carbidopa plus levodopa. Surgical history included bilateral total hip arthroplasty, prostate surgery, and cataract surgery, without anesthetic complications. There was no prior history of difficult airway management.

The American College of Surgeons National Surgical Quality Improvement Program (ACS-NSQIP) calculator estimated a 35.0% chance of serious complications, a 5.2% risk of cardiac events, and a 14.8% risk of mortality [5-7]. The Myocardial Infarction or Cardiac Arrest (MICA) score predicted a 4.5% risk of perioperative myocardial infarction or cardiac arrest [8], and the Preoperative Score to Predict Postoperative Mortality (POSPOM) score indicated a 12.8% risk of one-year mortality [9].

Coagulation analysis showed prothrombin time (PT) 16.0 seconds (normal value 11.4 s), activated partial thromboplastin time (aPTT) 36.0 seconds (normal value 29.4 s), international normalized ratio (INR) 1.45, aPTT ratio 1.20, and the patient’s anticoagulation was reversed with idarucizumab. Initial hemoglobin was 11.9 g/dL (normal range 13.0-17.0 g/dL), and blood typing was performed. The platelet count of 76000/μL (normal range 150-400 x 10³/μL) led to one pool platelet concentrate infusion before surgery. No other abnormalities were noticed in the complete blood count and metabolic panel.

**Figure 3:**
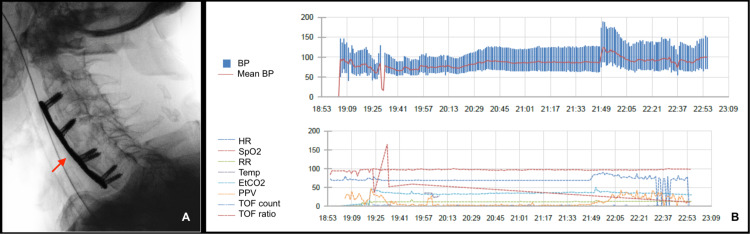
X-ray of the anterior cervical arthrodesis (A) and intraoperative monitoring tendencies (B) Arrow: C3-C7 anterior cervical arthrodesis; BP: blood pressure; HR: heart rate; SpO2: peripheral oxygen saturation; RR: respiratory rate; Temp: temperature; EtCO2: end-tidal carbon dioxide; PPV: pulse pressure variation; TOF count: train-of-four count; TOF ratio: train-of-four ratio

Surgical management was a C3-C7 anterior cervical arthrodesis (Figure 3A), under balanced general anesthesia. An awake nasal fiberoptic intubation was achieved with transitory remifentanil infusion, while maintaining manual and collar stabilization of the neck. No laryngeal edema or distortion was noted during airway management.

Intraoperative monitoring (Figure 3B) followed the American Society of Anesthesiologists (ASA) standards for basic anesthetic monitoring and included the pre-induction placement of an arterial line for invasive arterial blood pressure measurement and cardiac output assessment using waveform analysis (PulsioFlex™). Additionally, we used noninvasive hemoglobin monitoring (SedLine® SpHb). Arterial blood gas analysis showed pO2 91.2 mmHg (normal range 75.0-100.0 mmHg) with Fi02 35%, pCO2 of 29.9 mmHg (normal range 35.0-48.0 mmHg), and no electrolyte, lactate, or acid-base abnormalities. We monitored anesthetic depth with bilateral Patient State Index® and density spectral array (Sedline®) to titrate sevoflurane and remifentanil infusion. A General Electric® Healthcare adductor pollicis acceleromyography device was placed to manage neuromuscular blockade under rocuronium.

Fluid management was guided by dynamic hemodynamic indices (pulse pressure variation), and surgical blood loss was 100 mL. Total balanced crystalloid infusion was 2 L, although urinary output remained below 0.5 mL/kg/h. Intraoperative hypotension was managed with phenylephrine 100 μg boluses, followed by a norepinephrine infusion (0.1-1.6 μg/Kg/min) on a femoral central venous line. The mean arterial pressure target was set within 20% of the baseline value. Due to the conflicting surgical field and the patient's pacemaker non-dependent status, the multidisciplinary team decided to apply multifunction cardiac pads but not the magnet.

Postoperatively, the patient was transferred to the intensive care unit (ICU), sedated, and mechanically ventilated. Despite the high-risk profile, the patient showed a favorable clinical course, with gradual weaning from ventilatory support, complete neurological deficit recovery, and transfer to the orthopedic ward after 63 days. He was readmitted the following day to the ICU with a nosocomial respiratory infection causing type two respiratory failure, which led to the patient’s death 72 days after his initial admission to the hospital.

## Discussion

Cardiac amyloidosis is characterized by the infiltration of misfolded protein fibrils within cardiac tissue, leading to myocyte injury and restrictive cardiomyopathy [1-2]. In restrictive cardiomyopathy, myocardial stiffness causes impaired diastolic filling, which in turn results in fixed, reduced stroke volume, unresponsive to augmented preload or demand, and susceptibility to volume overload [3]. 

A common subtype is ATTRwt, which is disproportionately prevalent in elderly males [1,3]. Cardiac involvement includes, as seen in our patient, increased ventricular wall thickness, heart failure, conduction abnormalities, atrial fibrillation, and valvular dysfunction [2]. Atrial fibrillation is often an early indicator of cardiac involvement, particularly in ATTRwt [3]. Follow-up includes electrocardiography, cardiac biomarkers, and echocardiography [1], which helped us plan a safe anesthetic approach. Disease staging methods take into consideration NT-proBNP and troponin, eGFR, and functional status, but there are no risk stratification tools to predict anesthetic outcomes [1-2].

Heart failure pharmacologic regimes have to be managed to balance the risk of hypovolemia secondary to fasting and the risk of systemic congestion [3]. Although the evidence for SGLT2 inhibitors' cardioprotective effects in ATTR cardiac amyloidosis is still being studied [2], we should be aware of perioperative metabolic and electrolyte disorders that may arise secondary to our patients’ treatment. Point-of-care ultrasound to assess central venous filling may aid in preoperative volume status assessment [3]. As patients with cardiac amyloidosis are often on anticoagulation due to a high risk of thrombosis, independently of the CHADS-VASC score and even in the absence of atrial fibrillation [1-3], it is critical to discuss with experts the management of hematological disturbances. Disease-modifying treatments, like the transthyretin stabilizer tafamidis, can lead to blood cell line derangements [3], for example, thrombocytopenia, as seen in this case. Anticoagulation was reversed in our patient using dabigatran-specific monoclonal antibody therapy.

The anesthetic challenges associated with amyloidosis arise mainly from cardiovascular instability and complexities in airway management. Airway involvement presents more commonly in AL amyloidosis and includes macroglossia, subglottic stenosis, and frail airway tissue. Plan a step-by-step approach and ensure that adequate equipment is readily available for difficult airway management, as portrayed in our approach. Rapid sequence intubation may be necessary in these patients, for whom esophageal and gastroparesis are common [3]. In our patient, awake intubation with spontaneous ventilation was performed to preserve cervical spine stability.

Despite variable systemic manifestations, all variants of ATTR are heavily associated with cardiomyopathy and peripheral polyneuropathy [3]. Anesthetic agents may exacerbate hypotension due to vasodilation and myocardial depression, further impairing an already dysfunctional heart and autonomic system. In this case report, the presence of post-induction hypotension warranted the need for vasopressor support with noradrenaline, which should be preferred over phenylephrine for the management of hypotension in patients with pulmonary hypertension [10]. Cardiac output monitoring and dynamic indices allowed us to assess cardiovascular status and guide the use of fluid, vasopressors, and, if needed, inotropic support. Titrated induction with agents that minimize cardiac depression is advised, along with a pre-induction arterial line placement [3].

Due to the restrictive cardiomyopathy and diastolic dysfunction of cardiac amyloidosis, patients rely heavily on increased heart rate to maintain their cardiac output. Consequently, beta-blockers and calcium channel blockers are typically avoided, especially if there’s amyloid infiltration contributing to aortic valve stenosis. Conduction system dysfunction from amyloid deposition may lead to underlying atrioventricular nodal blocks, yet if present, bradycardia is usually resistant to anticholinergics due to parasympathetic denervation [3]. Standard pacemaker and cardiopulmonary resuscitation protocols should be followed [1-3]. Our approach followed the institutional protocol, which states that if patients are not pacing-dependent or if the electromagnetic interference source is at least 15 cm away from the pacemaker, reprogramming of the cardiac device or application of a magnet is not required. Implantable cardioverter-defibrillators for primary prevention are usually not present in patients with cardiac amyloidosis [1-2].

Other comorbidities, such as Brugada syndrome, require consideration in anesthetic planning, such as avoiding total intravenous general anesthesia with prolonged propofol infusion [11-12]. For patients with Parkinson's disease, propofol and most of the volatile anesthetic agents are safe; halothane should be avoided because of its potential arrhythmogenic effect and the risk of increased cardiac sensitivity to catecholamines. Our approach included an inhalational maintenance. Careful management of cardiovascular status also applies to patients with severe dysautonomia due to Parkinson’s disease, and prevention of postoperative nausea and vomiting should avoid antidopaminergic antiemetics [13].

The use of regional anesthesia can be appropriate in other surgical settings, but not without a previous complete neurological evaluation of existing deficits. Peripheral nerve blocks offer safer alternatives, as they avoid airway management and major hemodynamic effects. The risk of systemic hypotension is still present in neuraxial approaches; an intrathecal catheter placement may be considered [3].

Our approach is shaped by our hospital setting, team experience, and case-specific factors such as the type of surgery and emergency context. This case report aims to highlight the anesthetic challenges of managing a patient with cardiac amyloidosis, so these can be taken into consideration by peers in their medical practice. 

## Conclusions

A comprehensive preoperative evaluation and tailored intraoperative management are crucial in patients with amyloidosis to anticipate potential risks and to increase perioperative safety. Cardiac amyloidosis presents a significant perioperative challenge due to its association with restrictive cardiomyopathy, arrhythmias, conduction abnormalities, and valvular dysfunction, which predispose to profound hemodynamic instability. Preoperative assessment should include careful consideration of the patient’s medical history, current pharmacological therapy and its physiological implications, airway evaluation, comprehensive cardiac function documentation, and assessment of physical status. A tailored plan of the anesthetic technique, with avoidance of beta-blockers and calcium channel blockers, is essential to reduce myocardial depression. Advanced hemodynamic monitoring is useful in guiding vasopressor support and fluid therapy.
